# Challenges and opportunities in processing NanoString nCounter data

**DOI:** 10.1016/j.csbj.2024.04.061

**Published:** 2024-04-30

**Authors:** Jarosław Chilimoniuk, Anna Erol, Stefan Rödiger, Michał Burdukiewicz

**Affiliations:** a Clinical Research Centre, Medical University of Białystok, Białystok, Poland; b Institute of Biotechnology, Faculty Environment and Natural Sciences, Brandenburg University of Technology Cottbus - Senftenberg, Senftenberg, Germany; c Institute of Biotechnology and Biomedicine, Autonomous University of Barcelona, Barcelona, Spain

**Keywords:** NanoString, nSolver, mRNA, miRNA, nCounter, Background correction, Normalization, Differential expression

## Abstract

NanoString nCounter is a medium-throughput technology used in mRNA and miRNA differential expression studies. It offers several advantages, including the absence of an amplification step and the ability to analyze low-grade samples. Despite its considerable strengths, the popularity of the nCounter platform in experimental research stabilized in 2022 and 2023, and this trend may continue in the upcoming years. Such stagnation could potentially be attributed to the absence of a standardized analytical pipeline or the indication of optimal processing methods for nCounter data analysis. To standardize the description of the nCounter data analysis workflow, we divided it into five distinct steps: data pre-processing, quality control, background correction, normalization and differential expression analysis. Next, we evaluated eleven R packages dedicated to nCounter data processing to point out functionalities belonging to these steps and provide comments on their applications in studies of mRNA and miRNA samples.

## Highlights

• The R-based ecosystem for analysis of NanoString's nCounter data covers 11 different tools.
• We have categorized the steps of nCounter data processing into five distinct groups.
• Groups include pre-processing, quality control, background correction, normalization, and differential expression.
• NanoTube is the newest and the most robust R package for analysis of mRNA NanoString nCounter data via CLI or GUI.
• Since only NanoString nSolver facilitates ligation normalization, it remains the sole tool suitable for analyzing miRNA data.

## Graphical abstract

**Figure:**
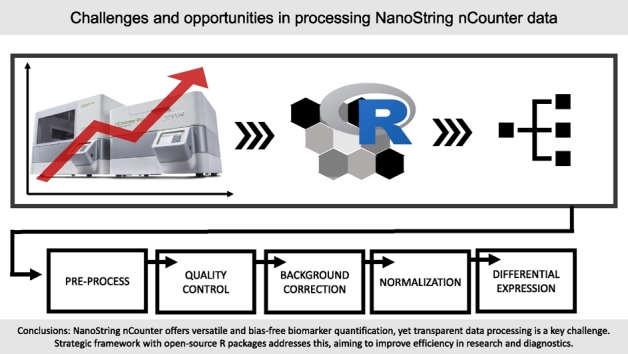

## 1 Introduction

Nucleic acid detection has many uses, including disease diagnosis, pathogen surveillance, and lab testing. However, choosing a detection method requires considering trade-offs between factors such as sensitivity, specificity, speed or cost. Among them, NanoString nCounter is one of the solutions tailored for the high-throughput (up to 800 targets) studies of even low-quality samples [45]. The nCounter permits the use of custom, relatively short (at least 100 bases) targets, so-called CodeSets. This versatility allows its use for various research applications in oncology, immunology, immuno-oncology, and neuroscience. An additional advantage lies in its simple and fast protocol, which minimizes the hands-on experimental time.

Thanks to nCounter's popularity, several competitive workflows exist for analyzing its data, including tools proposed by NanoString and others developed by the scientific community. In this review, we aim to survey and describe the existing R packages devoted to analyzing nCounter data. We will scrutinize their functionalities, categorize them into broader applications, and comment on how they complement the analytical pipelines the producer recommends.

### 1.1 NanoString nCounter workflow

**Fig. 1:**
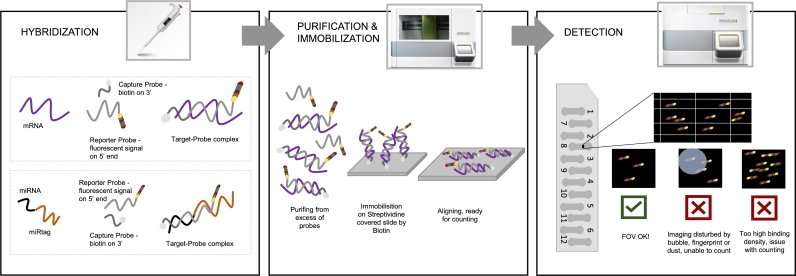
**NanoString's nCounter workflow.** The process consists of three steps. 1) Hybridization, where probes are hybridized to the target. 2) Purification & immobilization, during which excess probes are removed, and the sample is immobilized on a nCounter cartridge. 3) Detection, during this phase, barcoded reporter probes are identified.

NanoString nCounter relies on the hybridization of reporter and capture probes with the target (Fig. 1, Hybridization). mRNA/miRNA samples are mixed with probes, consisting of a reporter and a capture probe, and incubated overnight. For miRNA determination, the additional ligation of distinctive oligonucleotide tag (miRtag) is required to increase the specificity and sensitivity.

Reporter probes barcode their targets with a patterned color code at the 5' end to facilitate multiplex detection. The barcode consists of six positions with four distinct fluorescent colors permitting $4^{6}=4096$ possible color codes. However, due to kinetic and digital detection constraints, current technology limits the amount of simultaneously measured barcodes to less than a thousand DNAs, mRNAs, or miRNAs [13], [27].

Hybridized samples are then moved to Prep Station, a multichannel pipetting robot that removes excess probes that did not hybridize with the sample (Fig. 1, Purification & Immobilization). Next, the Prep Station distributes the sample to one of twelve available lanes on the nCounter cartridge. The capture probes immobilize the target into the Streptavidin-coated cartridge surface thanks to their biotin-labeled 3' end. Moreover, each lane contains positive control and negative control (probes without targets) as proposed by ERCC, External RNA Control Consortium. Optionally, in the case of miRNA profiling, lanes also contain ligation controls (the possible controls are described in subsection 2.1).

From Prep Station, cartridges are manually transferred to a Digital Analyzer, a multichannel epifluorescence scanner with a Charge-Coupled Device (CCD) camera. This equipment can simultaneously scan up to six cartridges, detecting the barcode of reporter probes (Fig. 1, Detection). The Digital Analyzer scans separately each lane and divides it into smaller regions known as fields of view (FOVs). The default number of FOVs per lane (280) can be increased to improve the dynamic range and level of sensitivity of the system.

The Digital Analyzer counts the number of optical features (fluorescent reporter probes) per FOV and tabulates the barcodes ignoring overlapping optical features. According to NanoString, the strict enforcement of this rule ensures that the molecular counts are from recognizable and distinguishable codes [22]. As an output, Digital Analyzer produces a Reporter Code Count (RCC) file containing the counts of specific barcodes, FOV count, FOV counted, and binding density for each lane. Additionally, it creates a Reporter Library File (RLF) reporting used target sequences and their barcodes. The counts of reporter probes are used to approximate the number of target molecules in the sample.

Both counted FOVs and binding density are critical for the quality control of the performed experiment. Counted FOVs represent the number of successfully imaged fields of view. The binding density measures the number of optical features (reporter probes) per square micron of the nCounter cartridge lane [7]. High values of binding density indicate that multiple optical features might be overlaping, which may lead to underestimated counts of optical features.

### 1.2 Detection and quantification of mRNA

Messenger RNA (mRNA) is the intermediate between the genetic information encoded in DNA and the synthesis of proteins. Thus, the mRNA expression levels provide valuable insights into the active genes in a given cell or tissue [29]. Therefore, there are multiple platforms and techniques to measure the level of mRNA expression, including reverse transcription followed by reverse transcription-quantitative polymerase chain reaction (RT-qPCR), microarray analysis, microbead-based assays, RNA-Seq, and northern blotting. These methods enable the quantification of mRNA levels, providing important insights into gene expression regulation at the transcriptional level [11], [36], [44].

For this application, the nCounter platform has shown a satisfying performance in benchmark studies, often outperforming other solutions. In a recent study, nCounter identified 88.4% of the transcripts and successfully revealed all the genes in the sample, whereas microarrays detected 82.6% transcripts and missed six of those genes [13].

When comparing nCounter and RT-qPCR for detecting type I interferon signature, both methods demonstrated similarly high sensitivity. However, nCounter proved to be more convenient due to its faster speed, simpler multiplexing, and easier automation [39].

Immunohistochemistry (IHC) and fluorescence in situ hybridization (FISH) are techniques commonly used to measure the level of different receptors and assess the response to breast cancer treatment. Recently, it was investigated whether nCounter or RT-qPCR could replace these tests. The study found that the results from the nanoString platform correlate better with the expression levels measured by IHC and FISH than qRT-PCR [21].

Both nCounter and RNA-Seq showed high precision, consistency, and reproducibility in discovering genes when compared for gene-specific intersample correlation validation. [5]. While nCounter does not offer a holistic insight into the whole transcriptome as RNA-Seq does, it is less stringent on sample quality, making it vital when analyzing low-grade clinical trial specimens. For this highly relevant context, nCounter's superior sensitivity, technical reproducibility, and robustness provide a substantial advantage over RNA-Seq [50].

### 1.3 Detection and quantification of miRNA

Small non-coding RNA molecules (miRNAs or micro RNAs), play a pivotal role in the post-transcriptional regulation of gene expression. They function as critical regulators of mRNA stability and translation, fine-tuning gene expression and influencing diverse cellular processes such as development, differentiation, and response to environmental cues [18].

Compared to other miRNA profiling techniques, nCounter did not prove to be superior in performance. In a large-scale test that included microarrays, nCounter, and NGS (New Generation Sequencing), the latter showed better performance, particularly in terms of dynamic range of detection and sensitivity [49]. Additionally, a more robust study that used extracellular RNA, crude biofluid, and synthetic RNA mixes independently confirmed the lower sensitivity of the nCounter platform, ultimately concluding that miRNA-Seq displays higher sensitivity than nCounter [16], [20].

However, nCounter still outperforms other techniques for archival formalin-fixed paraffin-embedded tissues. In a study utilizing hepatoblastoma samples, nCounter identified more miRNA targets than NGS and microarrays [8]. Moreover, nCounter usually exhibits specificity on par with NGS techniques, which is a highly desired feature in the clinical settings [49].

### 1.4 Prevalence of NanoString nCounter

**Fig. 2:**
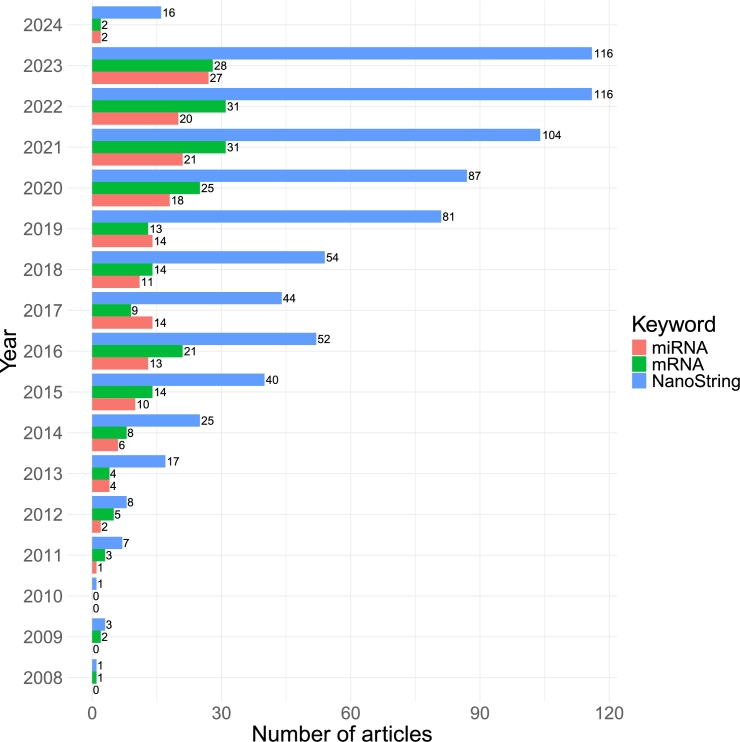
**NanoString's nCounter popularity.** Number of published articles associated with NanoString nCounter by year of the publication. The red, green and blue bars represent publications focused on mRNA, miRNA research or all publications referencing the NanoString nCounter technology.

The popularity of NanoString nCounter technology in studies on mRNA and miRNA has grown significantly over the last few years, as evidenced by the increasing number of related publications. This highlights the expanding role of this technology in influencing and helping advance various scientific disciplines. A PubMed search within article titles and abstracts was conducted on January 31, 2024, utilizing the following queries: 1) ‘nanostring AND (nsolver OR ncounter)’, 2) ‘(nanostring AND (nsolver OR ncounter)) AND mrna’ and 3) ‘(nanostring AND (nsolver OR ncounter)) AND mirna’ for articles centered around nCounter, which revealed a compelling uptrend (Fig. 2).

The increasing amount of articles referring NanoString nCounter technology in the context of mRNA and miRNA studies is evident until 2022. However, in 2022 and 2023, the number of publications remains almost constant, suggesting that the platform's popularity may stagnate in the coming years.

### 1.5 Advantages and limitations

Imaging technologies in biology and medicine often produce vast numerical data. For instance, in developmental biology, modern imaging techniques permit the visualization of numerous biological structures and processes, facilitating the quantification of physical parameters and cellular probes. In biomedical research they can facilitate quantitative metrics and molecular maps, thereby generating count data for further analysis [25], [53]. The unique capabilities and limitations of nCounter provide it with a unique place between other imaging technologies.

nCounter is unsuitable for biomarker discovery as it employs approximately 800 preselected genes, which may limit its use in discovering new target genes. This limitation is particularly evident when compared with microarray assays or the ability to sequence whole genomes or transcriptomes provided by NGS. Besides, nCounter may be less sensitive to minor changes in gene expression compared to other methods, potentially leading to a narrower range of gene expression and reduced sensitivity to detect low-abundance RNA species in some cases [3]. In contrast, the quantitative PCR (qPCR) and the digital PCR (dPCR) are more appropriate methods because they use enzymatic amplification processes and are, therefore, more sensitive than the hybridization-based nCounter technology.

The nCounter is predominantly used in assays to quantify RNA species, particularly mRNAs. Unfortunately they account for only ∼5% of total RNA in the sample, limiting the number of binding events [1]., making the platform, in theory, dependent on larger RNA inputs. This relatively low abundance of mRNA that could be bound by the probes makes the platform theoretically dependent on more significant sample inputs.

The higher sample volumes are more convenient in pipetting because they mitigate possible volume inaccuracies. However, from personal experience and communication with peers, it is well-accepted that smaller amounts of starting material are needed (circa 100 ng of total RNA or lysate from 10,000 cells). These are similar quantities to qPCR and dPCR but less than RNAseq, requiring more than 1 μg of high-quality total RNA for most assays. Therefore, these technological requirements should be considered when choosing a transcriptomic technology for a specific research or clinical application [35], [28].

## 2 nCounter data processing

### 2.1 Sources of bias and noise in nCounter data

The noise in nCounter data stems from various factors, including technical and biological variation, as well as limitations of the normalization process. Technical variation in nCounter data can result from factors such as batch effects over time, RNA preservation methods, and probe-specific differences in background.

Accurate estimation of probe background and biological variation, including sample-specific differences, is crucial for interpreting expression data. The nCounter includes several negative control probes in each CodeSet to estimate background values in the experiment. However, these controls may not always capture minor probe-specific differences in the background or biological variation for every probe, which could potentially introduce noise.

Normalization methods tackle the bias-variance trade-off, where removing a systematic bias may amplify the effect of the random errors. The incorrect normalization prevents accurate quantification of gene expression levels and may increase noise in the data [31]. To counteract this, nCounter offers several distinct positive controls: positive normalization factors, spike-ins, and housekeeping genes (HK).

The most basic method of normalization utilizes positive control normalization factors based on the ERCC positive control probes to detect and account for technical variation [34]. Although the default acceptable range values are 0.3-3.0, there are data processing frameworks that utilize samples outside of it [4].

Spike-ins serve two primary purposes in genomic data preprocessing, including RNA hybridization assays. Firstly, they act as a quality control parameter, confirming the dynamic range and quantitativeness of the sequencing experiments. Secondly, spike-ins enable absolute normalization, converting read counts to copies per microliter (μl) of total RNA and reducing bias originating from RNA variation. These spike-ins are RNA transcripts of known sequence and quantity, designed to bind to RNA molecules with matching sequences, allowing for the calibration of measurements in various genomic analyses such as microarray experiments, RT-qPCR, and RNA-Seq [24]. In nCounter experiments, spike-ins are used as positive controls, and probes without targets are used as negative controls.

Housekeeping or reference genes refer to a set of genes that are constitutively expressed at relatively stable levels across different cell types and experimental conditions. These genes serve as reference controls in gene expression studies to normalize expression levels of target genes, ensuring accurate quantification of gene expression. They can also be used to monitor the progress of the experiment, serving as reporters of technical problems. In this sense, normalization using HK genes helps to correct for variations in RNA input, assay efficiency, and other technical factors, thereby reducing noise introduced by these sources of variation [27], [26].

Over- and underdispersion are common sources of noise in image-based count data, where the variance differs significantly from the mean, breaking the assumption of the Poisson distribution. Due to the way that Digital Analyzer processes optical features (e.g., ignoring overlapping features), modeling such data using the Poisson distribution might lead to significant bias [9]. Therefore, it is recommended to use more flexible distributions, such as the Gamma distribution [52].

### 2.2 NanoString nSolver software for nCounter data processing

NanoString provides users with nSolver, a graphical user interface (GUI) software [34]. The software is tailored to analyze data generated by the nCounter system, aiming to streamline the complex process of data analysis. It offers a limited toolkit to perform quality control, normalization, and downstream analysis of gene expression data, thereby reducing the complexity of data analysis and restricting it to methods proposed by the producer.

Nevertheless, the scientific community has supplemented proprietary software by developing versatile and open-source tools that provide additional processing capabilities and visualization methods. The statistical programming language R [40] is widely used in bioinformatics, particularly for reproducible analysis related to gene expression, encompassing mRNA and miRNA quantification via dPCR, microarrays and NGS. Its extensive library of statistical and graphical packages, along with integration with Bioconductor, a rich repository of R packages for computational biology and bioinformatics [15], makes it well-suited gene expression data analysis. A large and active community of users and developers constantly create new tools and packages [15], [14], [36], [46]. Within them, we can find packages specifically focused on the processing of nCounter data (Table 1).

**Table 1:** **R packages for NanoString nCounter data processing.** The *Package name* column contains names of the R packages. The *Peer-reviewed* column informs if the package was published in a peer-reviewed journal, and the *DOI* column links it to the appropriate doi identifiers. The *Source* column includes links to the respective packages, and the *Last update* column indicates whether development of the package is ongoing and specifies the date of the last revision. The *GUI* column denotes the presence of a Graphical User Interface, if applicable. Packages are sorted by the date of the last update.

Package name	Peer-reviewed	DOI	Source	Last update	GUI
NACHO	Yes	10.1186/1471-2105-12-479	https://cran.r-project.org/package=NACHO	12 Jan 2024	Yes
nanostringr	No	-	https://cran.r-project.org/package=nanostringr	15 Aug 2023	No
NanoTube	Yes	10.1093/ bioinformatics/btac762	https://bioconductor.org/packages/NanoTube	8 Mar 2023	Yes
NanoString- NCTools	No	-	https://bioconductor.org/packages/NanoStringNCTools.html	25 Jan 2023	No
RCRdiff	Yes	10.1002/ sim.9250	https://github.com/canx2021/RCRdiff	17 Jun 2021	No
NanoString- Norm	Yes	10.1093/ bioinformatics/bts188	https://cran.r-project.org/package=NanoStringNorm	11 Sep 2020	No
NanoStriDE	Yes	10.1186/1471-2105-12-479	https://github.com/cbrumbau/NanostriDE	3 Mar 2018	No
RCRNorm	Yes	10.1214/19-aoas1249	https://cran.r-project.org/package=RCRnorm	22 Feb 2018	No
NanoString- Diff	Yes	10.1093/ nar/gkw677	https://bioconductor.org/packages/NanoStringDiff	5 Feb 2018	No
nanoR	No	-	https://github.com/KevinMenden/nanoR	23 Oct 2017	No
NAPPA	No	-	https://cran.r-project.org/package=NAPPA	3 Mar 2015	No

**Fig. 3:**
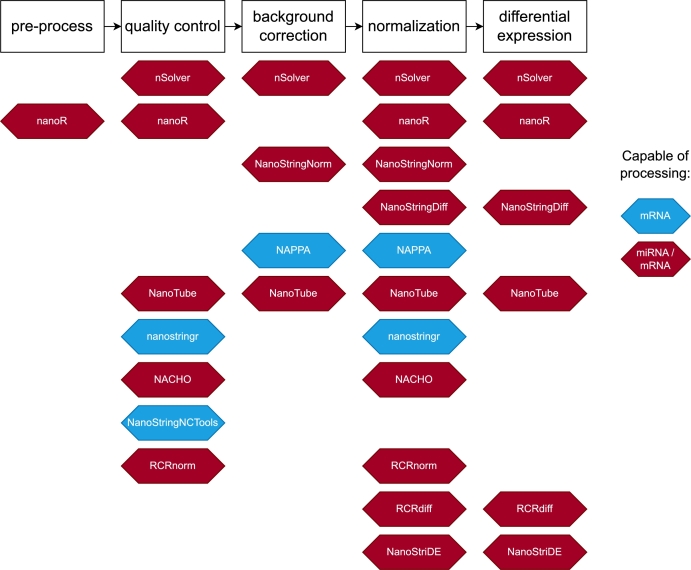
**Elements of analytical workflow in NanoString data processing R packages.** The figure presents the sequential steps involved in NanoString data analysis. White rectangles represent elements of the analytical workflow, beneath which are packages that contain functions categorized to this phase. Each package is color-coded to indicate the type of RNA it can process, which was mentioned in the text or the RNA data was used as an example in the vignette.

We list eleven R packages designed for processing nCounter data (Table 1). They encompass five main workflow elements: pre-processing, quality control, background correction, normalization, and differential expression analysis (Fig. 3). Notably, none of the packages cover all these elements, while nSolver software covers only four. Moreover, eight packages are described as working efficiently with mRNA and miRNA data, and two are exclusively dedicated to mRNA analysis.

### 2.3 Data pre-processing

For the sake of this article, we define data pre-processing as background correction or normalization occuring before any quality control. The nCounter guidelines do not discuss this step and almost all tools designed for nCounter data analysis skip it.

The only tool that examinates this aspect of the analytical workflow is the nanoR package [30], which uses data pre-processing background correction, positive control normalization or both. Background correction involves computing the mean counts of all background probes (negative control probes), adding two standard deviations to this mean value and subtracting it from the samples. Positive control normalization utilizes spike-ins, with their geometric mean calculated to determine positive control factors.

### 2.4 Quality control

Raw data obtained from nCounter must undergo quality control (QC) procedures. QC involves verifying the parameters of the measurements to ensure their reliability for subsequent data analysis. In this step, we flag the samples that do not fulfill specific criteria: imaging, binding, ligation, positive control, positive control linearity, and limit of detection [32]. Users might decide to remove samples that fail to positively address one or more of these criteria from the next steps of data processing.

Imaging QC relies on FOV analysis, which assesses the ratio of FOV count and FOV counted. Optimal performance is achieved when the ratio exceeds 75% [32]. This parameter is taken into consideration in by nSolver, NACHO [7], NanoStringNCTools [38] and nanostringr [48].

Another important parameter is Binding QC, which evaluates binding density. The recommended range differs depending on the device (0.1–2.25 for MAX/FLEX and 0.1–1.8 for SPRINT instruments). If the maximum overlap of reporters on the nCounter cartridge lane is outside these recommended ranges, this could reflect inaccurate counting of optical events [32]. Binding QC is integrated into nSolver, nanoR, NanoStringNCTools, and NACHO.

Ligation QC is exclusive to nSolver software. It utilizes six short synthetic RNA constructs, three of which undergo ligation, acting as a positive ligation control, and the remaining three functions as ligation-negative controls. The ligation is crucial to properly attach miRtag to miRNA samples (described in the subsection 1.1). The lack of the proper ligation implies that the measured level of miRNA might be lower than in reality. During ligation QC, counts of ligation controls are compared with the background level. Negative controls should be lower and the positive control higher than the background [32].

Positive control QC is employed by nSolver software, NanoTube [10], nanoR and NACHO. This QC flags samples that exhibit a more than three-fold count difference than the mean of all positive control samples [32]., overall excluding samples that contain relatively few optical features.

Positive control linearity QC is determined by analyzing the linear relationship between $\log_{2}$-transformed counts of positive control samples and their concentrations. The resulting $R^{2}$ should not fall below 0.95 [32]. This QC is used by nSolver software, NanoTube, nanoR, NanoStringNCTools and nanostringr. The lack of linearity between concentration and the number of optical features suggests that nCounter might not be able to reliably estimate the real concentration of a target molecule in the sample.

The level of detection (LOD) QC assesses the difference in counts between negative controls and the background. The negative control probe with the lowest concentration should generate raw counts at least two standard deviations higher than the mean of the negative control probes [32]. This QC indicates if the dynamic range of the system is sufficient. LOD QC can be found in nSolver software, nanoR, NanoStringNCTools, NACHO and nanostringr.

Furthermore, nanostringr, and NanoStringNCTools propose assessing HK genes using their mean logarithmic expression level as a QC metric. This value should not exceed a 10-fold difference or fall below 0.1, indicating significantly lower counts than the sample's average. This QC indicates if the HK genes exhibit sufficient levels to be reliably used for normalization.

### 2.5 Background correction

Background correction removes background signals stemming from non-specific binding of probes, sample impurities, or technical artifacts. Background correction is crucial in experiments where the low-expressing targets are prevalent, leading to occasional false positives in the counts for each target [4]. Although the negative controls play an essential role in estimating the size of the background correction, there are two main strategies of its implementation.

The first method for background correction is background thresholding, where all counts that fall below the threshold level are adjusted to match it, making a threshold level a minimum amount of counts that nCounter could reliably measure. Effectively, background thresholding preserves the distribution counts of samples with the signal stronger than the background.

The threshold level can be defined using a set of arbitrary functions. The most common is arithmetic means of negative control counts. Other alternatives apply mean plus two standard deviations of negative control counts, maximum negative control counts or even a user-defined constant. This method is preferred in samples with low-level expression to enhance sensitivity but at the cost of potentially including false positives. The function can be found in the nSolver, NanoTube and NanoStringNorm [51].

The other approach for background correction is background subtraction, which relies on subtracting the background value from the obtained counts. Samples with fewer counts than the background value are usually treated as having no counts. Thus, background subtraction affects the overall distribution of counts by reducing the mean.

Background subtraction can be done by using mean counts of negative control, mean counts from the blank lane or a user-defined value. Although this approach is not recommended by NanoString [33], it is implemented in nSolver and in independent software as NanoTube and NanoStringNorm. A unique variant of background subtraction relies on using mean counts of sequence tags absent from the assay instead of the negative control, as employed by NanoStriDE and NAPPA [19].

NAPPA is the only method employing a more advanced statistical model for background correction. It assumes that counts follow a truncated Poisson distribution adjustment, restricting observed positive counts to a certain threshold (background). The model estimates the threshold using the distribution of positive counts, potentially allowing a more fine-grained threshold estimation. However, the extent to which the truncated Poisson distribution accurately reflects NanoString nCounter data behavior remains unverified.

### 2.6 Normalization

During the normalization process, counts of optical features in each sample are adjusted by a defined factor. The proper normalization mitigates unwanted variation arising from technical replicates and potential RNA degradation resulting from sample fixation. Thus, a proper normalization is vital to compare samples measured on different cartridges [12], [31], [32].

Positive control normalization involves determining the mean of positive control samples for each assay lane and computing the global arithmetic mean of these lane-specific means. The global arithmetic mean is then divided by the geometric mean of each lane, resulting in a lane-specific normalization factor. Subsequently, gene counts are normalized by multiplying them by their corresponding lane-specific normalization factor. This method is implemented in nSolver, NanoStriDE [6], NAPPA, NACHO and nanostringr.

Alternatively, nSolver can use ligation control samples instead of positive controls. This is a recommended normalization method for miRNA samples [33]. It is important to notice that other tools do not offer this possibility, even when they outright define themselves as designed for miRNA data (nanoR, NanoStringNorm, NanoStringDiff, NanoTube, NACHO, RCRnorm, RCRdiff, NanoStriDE).

Similarly to qPCR, normalization may employ HK genes, as they vary only according to how much sample RNA was loaded. Therefore, utilizing HK normalization is recommended when sample input variability is expected. As a normalizing factor, it is possible to use the geometric mean, total sum, or the top geometric mean (the geometric mean of the HK genes with the highest count). Normalization using the counts of HK genes can be found in nSolver software, nanoR, NAPPA, NACHO and nanostringr. For miRNA assays, the geometric mean is recommended. This is implemented in NanoStringNorm and NanoTube (which use functionalities of NanoStringNorm to perform the normalization).

NanoR as well as nSolver provide two normalizations: global and top100. Global normalization utilizes the sum of all the endogenous gene probe counts for each lane excluding positive and negative controls. This normalization is recommended when a relatively small fraction of genes is expected to be differentially expressed. In contrast, the top 100 normalization is designed for situations when only a few genes in the assay are expressed above the threshold level. Top 100 normalizing factor utilizes the geometric mean from the highest expressed one hundred genes.

NanoStringNorm also covers the code-count normalization. It normalizes technical assay variation by adjusting each sample to a factor, involving summarized code counts using mean, median, sum, or geometric mean relative to all samples. Moreover, it can use probe correction factors by multiplying them by the maximum value for each sample and subtracting the result from the raw count. This is the recommended method of normalization for highly multiplex samples.

NAPPA adjusts each lane based on the counted FOV. This should allow for comparing cartridges with drastically different numbers of successfully imaged fields of view, provided that the average number of optical features per FOV is similar (which might be untrue). As the low amount of counted FOV is usually an indication of incorrectly prepared samples, we would recommend repeating the experiment instead of utilizing this normalization method.

NanoStringNorm offers additional normalization methods such as quantile, zscore, rank.normal, vsn. The quantile normalization, via quantile, is a method to make the distributions of different samples (or datasets) identical. It ensures that the distribution of the intensities for a given probe is the same across different samples. Z-score normalization, via zscore, is a method that scales and centers the data so that it has a mean of 0 and a standard deviation of 1. The normalization via rank.normal transforms the data to follow a normal distribution. It is particularly useful when the data does not meet the assumptions of normality. Variance Stabilizing Normalization (vsn) is a method that stabilizes the variance across the intensity range, making it more consistent and suitable for further analysis, particularly for count data. In general, all these methods facilitate comparing targets with different distributions of optical features.

NanoStriDE utilizes DESeq normalization estimating effective library size, called the size factors vector, to balance the number of samples for comparability. This is a non-parametric normalization that ensures that the variance is similar among all samples [2].

NanoTube incorporates the RUVg [42] and RUV-III (Remove Unwanted Variance III) [31] methods. RUVg uses HK genes or spike-ins to normalize the data, while RUV-III (Remove Unwanted Variance III) is designed to normalize data leveraging technical replicates. Moreover, in the absence of real replicates, pseudoreplicates from pseudosamples will be generated increasing the applicability of this method. RUV-III returns log-transformed counts.

RCRnorm [23] and RCRdiff [54] adopt the Bayesian model to normalize gene expression and estimate parameters across samples. The model incorporates information from all aspects of the experimental design and removes biases, simultaneously eliminating unrealistic assumptions about HK genes and offering greater interoperability.

NACHO uses a generalized linear model with a negative binomial family provided by NanoStringDiff [52] to determine the positive scaling factor. This is done by first calculating the average of all sample slopes. Then, the average slope is divided by the individual slope of the respective samples.

### 2.7 Differential expression

Differential expression (DE) analysis is employed to identify genes that exhibit variations in expression levels among various conditions or are otherwise linked to specified predictors or responses [47], [52].

The primary approach for DE analysis often employs a student's t-test, which is applied to logarithmically transformed count data. This transformation helps meet the t-test's assumption of normal distribution, while the heteroscedastic t-test further allows for unequal variance among groups. The resulting output provides a p-value, with genes considered significant if the nominal p-value is less than 0.05. A lower p-value indicates stronger evidence that a gene exhibits distinct expression levels between the two groups. Student's t-test for DE is used in nSolver software, nanoR and NanoStriDE.

NanoTube and nanoR for the differential expression employ limma (linear models for microarray data) package [43]. It offers robust tools for differential gene expression and DNA methylation data analysis, incorporating functions like quantile normalization, z-score normalization, and variance stabilizing normalization (VSN).

Moreover, limma integrates multiple testing correction. This is essential in genomics analysis as it addresses the perk of numerous statistical tests simultaneously, which inherently increases the likelihood of erroneously identifying significant results purely by chance. Effectively, this results in minimizing false discovery rate (FDR), ensuring high reliability and reproducibility of research findings [17].

DESeq [2], implemented in NanoStriDE package also employs multiple testing correction. NanostriDE utilizes different methods to validate the statistical significance of the DE depending on the distribution of the data. For a negative binomial distribution with two conditions, it uses DESeq method, while for three or more one-ways, it employs ANODEV, from the DESeq package.

NanoTube and NanoStringDiff [52] utilize a generalized linear model (GLM) within the negative binomial family to compensate for over- and underdispersion commonly found in count distributions. The approach incorporates positive controls, negative controls, and HK genes to normalize the data. Next, the empirical Bayes shrinkage approach is used to estimate the dispersion parameter and a likelihood ratio test to identify differentially expressed genes.

Lastly, RCRdiff utilizes Bayesian LASSO to identify differentially expressed genes. This method does not require normalization, streamlining the data processing pipeline and removing the risk of introducing artifacts during this step.

## 3 Conclusions

One of the standout advantages of the NanoString nCounter technology is its capability for absolute quantification of biomarkers. Unlike other techniques, this method does not involve any amplification steps, theoretically minimizing bias in experimental outcomes [37]. This unique characteristic makes the NanoString nCounter a well-balanced approach between amplification-dependent methods like qPCR/dPCR and high-multiplex approaches like microarrays and NGS.

It is important to acknowledge that despite the absolute quantification provided by NanoString nCounter technology, bias cannot be entirely ruled out. Indeed, this technology's inherent sensitivity to the variability of gene expression counts arising from sample acquisition, storage, and preparation underscores the necessity of making informed decisions regarding analytical pipelines. Consequently, selecting appropriate analytical methodologies is imperative to ensure the data obtained is robust and reliable.

Our work described the nCounter data processing workflows using five essential steps: data pre-processing, quality control, background correction, normalization, and differential expression analysis. Next, we surveyed eleven relevant open-source R packages, assigned their functionalities to these steps and assessed their ability to analyze mRNA or miRNA data. This methodical approach aims to provide a uniform description of NanoString nCounter data analysis, making it easier to compare different tools.

It is also important to highlight that these tools differ in their accessibility. Besides nSolver, only NACHO and NanoTube offer GUIs. This aspect greatly enhances their usability, but could also negatively affect the reproducibility of the conducted analysis [41].

In conclusion, we recommend using NanoTube to analyze mRNA data as it is the most robust of the proposed tools and includes a working GUI. Since only nSolver implements ligation control for normalization, it remains the only tool to process miRNA data properly. Finally, RCRdiff provides the only method for differential expression analysis that does not require any normalization.

## CRediT authorship contribution statement

**Jarosław Chilimoniuk:** Writing – review & editing, Writing – original draft, Visualization, Methodology, Formal analysis, Data curation, Conceptualization. **Anna Erol:** Writing – review & editing, Conceptualization. **Stefan Rödiger:** Writing – review & editing, Conceptualization. **Michał Burdukiewicz:** Writing – review & editing, Writing – original draft, Supervision, Project administration, Funding acquisition, Data curation, Conceptualization.

## Declaration of Competing Interest

The authors declare that they have no known competing financial interests or personal relationships that could have appeared to influence the work reported in this paper.
